# High Performance DNA Probes for Perinatal Detection of Numerical Chromosome Aberrations

**DOI:** 10.4172/2379-1764.1000155

**Published:** 2015-12-03

**Authors:** Kalistyn H Lemke, Jingly F Weier, Heinz-Ulrich G Weier, Anna R Lawin-O’Brien

**Affiliations:** 1 Life Sciences Division, University of California, E.O. Lawrence Berkeley National Laboratory (LBNL), Berkeley, USA; 2 Dermatopathology Service, School of Medicine, University of California, San Francisco, USA; 3 Centre for Fetal Care, Queen Charlotte's and Chelsea Hospital, Imperial College Healthcare, London, UK

**Keywords:** Pregnancy, Chromosomal imbalance, Aneuploidy, Perinatal diagnosis, Molecular cytogenetics, Fluorescence *in situ* hybridization (FISH), DNA probes

## Abstract

Human reproduction is a tightly controlled process of stepwise evolution with multiple, mostly yet unknown milestones and checkpoints. Healthy halpoid gametes have to be produced by the parents, which will fuse to form the diploid zygote that implants in the female uterus and grows to become first an embryo, then a fetus and finally matures into a newborn. There are several known risk factors that interfere with normal production of gametes, spermatocytes or oocytes, and often cause embryonic mortality and fetal demise at an early stage. Yet some embryos with chomosomal abnormalities can develop beyond the critical first trimester of pregnancy and, while those with supernumary chromosomes in their hyperdiploid cells will be spontaneously aborted, a small fraction of fetuses with an extra chromosome continues to grow to term and will be delivered as a liveborn baby.

While minor clinical symptoms displayed by children with trisomies are manageable for many parents, the burden of caring for a child with numerical chromosome abnormalities can be overwhelming to partners or individual families. It also poses a significant financial burden to the society and poses ethical dilemma.

In this communication, we will review the progress that has been made in the development of molecular techniques to test individual fetal cells for chromosomal imbalances. We will focus our discussion on the direct visualization of chromosome-specific DNA sequences in live or fixed specimens using fluorescence in situ hybridization (FISH) and, more specifically, talk about the groundbreaking progress that in recent years has been achieved towards an improved diagnosis with novel, chromosome-specific DNA probes.

## Mini Review

Numerical chromosome aberrations are rarely compatible with early human development and life. Most commonly, chromosomally imbalanced human zygotes or embryos carry chromosomal monosomies or trisomies leading to either failed nidation or fetal demise. Published estimates state that as many as half of the 15–20% of recognized pregnancy failures are due to numerical chromosome aberrations [1]. A few well known exceptions are embryos carrying an extra chromosome 13, 18, 21, X or Y, which lead to phenotypical abnormalities and clinically recognizable symptoms [1–6].

However, incidences of trisomy occur disproportionately among the 22 human autosomes. Studies karyotyping thousands of live birth or spontaneous abortuses, sometimes referred to as ‘product of conception (POC)’, show that trisomies involving chromosome 16 are the by far most common abnormality and are found in 31% of spontaneous abortions [1] compared to trisomy 13 or 21 found in only 4.1% and 10.5% of spontaneous abortions. Chromosome 16 trisomy occurs in 1–2% of all human conceptions and is thus most common autosomal trisomy found in first trimester miscarriages [7,8].

Studies of human preimplantation embryos at the day 3-stage could demonstrate a maternal age dependent increase in the number of embryos carrying cells with an extra chromosome 16 [9] providing further evidence that most trisomy 16 pregnancies originate as a consequence of a maternal meiosis I non-disjunction [10] and are generally not compatible with life [1,11].

However, some embryos which survive early in utero carry a rare trisomy 16 mosaic aberration, containing both euploid and trisomic cell lines with abnormal expression of imprinted genes [12,13]. Such cases include true mosaics, cases with confined placental mosaicism (CPM) and uniparental disomy (UPD) [7].

A trisomy 16 mosaicism is usually caused by a remarkable process termed ‘trisomy rescue‘, where loss of a chromosome 16 in one of the trisomic cells of the early embryo results in a euploid cell line. The final distribution of trisomy 16 cells in the placenta and the fetus depends on the embryonic stage when trisomy rescue occurs and either one of the two maternal chromosomes or the paternal chromosome can be lost [7,14]. When a trisomy 16 conceptus is rescued, the result may be maternal uniparental disomy 16 (UPD(16)mat), i.e, both homologues of chromosomes 16 are inherited from the mother [15,16] and a lifeborn child may have a distinct phenotypic effect [7,14,17]. While, a significant number of fetuses with prenatally diagnosed mosaic trisomy 16 have a good outcome with a milder phenotypical appearance [6,18], the mosaic trisomy 16 has also been associated with a severe pregnancy complication called ‘preclampsia‘ [13] emphasizing a need for rapid and accurate genetic analysis of fetal chromosomes as a further relevant tool for counselling the mother [10,11,14,19,20].

For more than two decades, our laboratories and others have been involved in the design of genetic tests analyzing the karyotypes of human sperm, oocytes, preimplantation embryos, fetal cells and tumor specimens based on fluorescence *in situ* hybridization (FISH) [3,21–26].

The FISH technique is based on hybridization of non-isotopically labeled nucleic acid probes and detection by fluorescence microscopy [27]. Sources of DNA probes can be any modified oligonucleotide or chunks of cloned DNA identified as chromosome- or gene-specific sequence. The goal of DNA probe optimization is to increase probe specificity and signal intensity. While small synthetic oligonucleotide probes have the advantage of rapid diffusion and thus shorter hybridization times, the small number of fluorescent moieties bound to intracellular targets often results in weak signals.

An ideal, high performance DNA probe is highly chromosome-specific and works well with a spectrum of biological specimens ranging from archival to fresh samples that may have undergone complex aging and fixation procedures [28]. Our laboratories and others have worked with a variety of cloned or PCR-amplified DNA sequences targeting tandemly-repeated pancentromeric clusters of alpha satellite DNA [21,29–32] for chromosome enumeration. Early probes were often cloned in plasmids which allow inserts in the kilobase (kb) range to be stably propagated [33,34]. However, some alphoid DNA probes face limitations of use in cases where existing heteromorphisms lead to one strong and one very weak signal, the latter of which might be easily missed [35,36].

The family of short DNA satellite repeats II/III is an attractive, large hybridization target for chromosome enumeration, even in the presence of heteromorphisms [35], but remains limited to just a handful of human chromosomes such as chromosomes 1, 9, 16 and Y. The plasmid clone pHUR98 is an example of a successfully used chromosome 16 satellite II/III DNA repeat probe [37–39].

We gained extensive experience preparing FISH probes from yeast artificial chromosome (YAC) clones or P1 clones [40–43]. However, existing physical maps for these clones do not cover the heterochromatic regions of the human genome and most probes will target single copy sequences which result in weaker signals.

**Figure 1:**
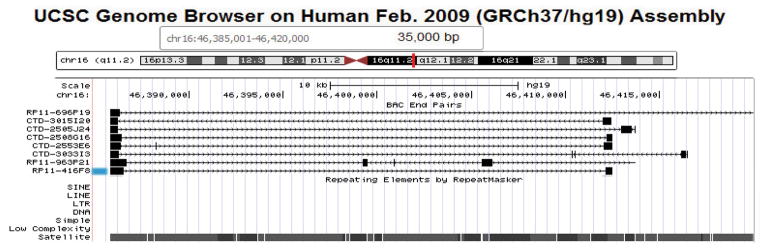
The Graphic User Interface (GUI) of the UC Santa Cruz online Genome Browser indicating the position of BAC clone RP11-416F8 (indicated by the blue mark on the left) along the draft sequence of human chromosome 16.

Other approaches for chromosome enumeration have been described such as comparative genomic hybridization [44], but none of them can compete with the above describe FISH and direct visualization in terms of speed, low complexity and cost. We recently investigated the use of bioinformatics tools and mining of data in existing databases. We chose to search the genome database at UC Santa Cruz [45], which shows alignments of paired end-sequenced bacterial artificial chromosome clones (BACs) [46,47] with the provisionally finished draft of the human genome sequence [48]. In our most recent approach to prepare high performance DNA probes that supersede preexisting probes with regard to probe specificity and signal-to-noise ratios, we attempt to identify BAC clones from the pericentromeric regions of different chromosomes that are free of non-chromosome-specific short or long interspersed repeat sequences (SINE’s, LINE’s) and contain ‘pure’ satellite DNA (Figure 1) [49–51].

**Figure 2:**
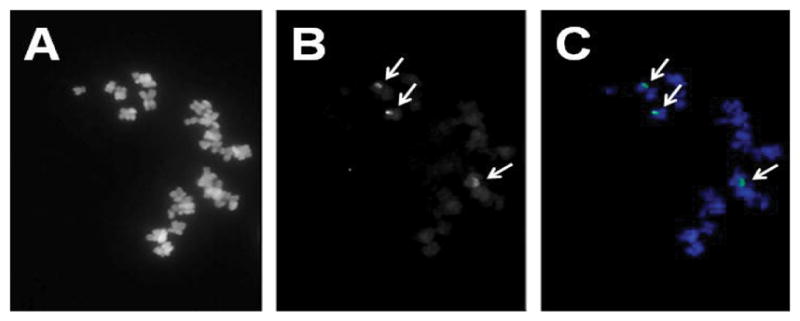
FISH result using a biotinylated probe prepared from BAC clone RP11-416F8. A) DAPI image showing the DNA/metaphase chromosomes; B) Three chromosome-specific green fluorescence signals (arrows) after staining with avidin-FITC; C) Overlay of the DAPI and FITC images.

As the example in Figure 1 illustrates, the alignment of paired end-sequences from BAC clone RP11-416F8 suggests an insert of about 26.678 kb of human DNA mapping to chromosome 16, band q11.2 [51]. The insert is predicted to be comprised entirely of satellite DNA. We isolated the BAC DNA and labeled it with biotin using a Bioprime kit (Life Technologies, La Jolla, CA). The probe, when hybridized to metaphase spreads prepared from the fibroblast cell line WI-38 and stained with avidin-FITC following our published protocol [24], showed two very bright, specific signals per cell (arrows in Figure 2B) which were easy to count in the microscope by eye. Other probes, which have been prepared using a similar bioinformatics approach for chromosomes 10, X and Y can easily be combined with this chromosome 16-specific probe [50–52].

The fact that the WI-38 human diploid cell line was derived by Leonard Hayflick from normal female embryonic (3 months gestation) lung tissue in 1962 does not necessarily imply that the particular batch of WI-38 cells used in our studies was diploid [53]. As Sigma Aldrich, a major supplier of WI-38 cells for research and vaccine production describes the cells on their web site as 'Fibroblast-like, 2n=46, diploid except at high passage number', karyotype alterations have to be anticipated at high number of passages [54]. The observation of three chromosome 16-specific signals in WI-38 cells matches the primary goal of our technical developments, i.e., an assay able to detect trisomy 16 in fetal tissues.

In summary, despite the fact that most of the human heterochromatin remains unchartered territory, simple data mining approaches can identify potential DNA probes for chromosome enumeration that are easy and inexpensive to prepare by the less experienced laboratory and result in FISH signals of unprecedented specificity and intensity.

## Abbreviations

BAC: Bacterial Artificial Chromosome
CPM: Confined Placental Mosaicism
DAPI: 4,6-Diamino-2-Phenylindole
FISH: Fluorescence *In Situ* Hybridization
FITC: Fluorescein Isothiocyanate
UPD: Uniparental Disomy
